# Association of serum immunoglobulin G (IgG) levels against two periodontal pathogens and prothrombotic state: a clinical pilot study

**DOI:** 10.1186/1477-9560-8-16

**Published:** 2010-11-04

**Authors:** Sergio Bizzarro, Elena A Nicu, Ubele van der Velden, Marja L Laine, Bruno G Loos

**Affiliations:** 1 Department of Periodontology, Academic Center for Dentistry Amsterdam (ACTA), University of Amsterdam and Vrije University, Amsterdam, The Netherlands; 2 Department of Oral Microbiology, Academic Center for Dentistry Amsterdam (ACTA), University of Amsterdam and Vrije University, Amsterdam, The Netherlands; 3 Department of Periodontology, Catholic University Leuven, Leuven, Belgium

## Abstract

**Objective:**

Periodontitis is associated with cardiovascular diseases (CVD). In our previous studies a prothrombotic state has been observed in periodontitis, which contributes to the risk of CVD. The aim of this study was to investigate whether serum IgG levels against *Aggregatibacter actinomycetemcomitans (Aa) *and *Porphyromonas gingivalis (Pg) *in periodontitis were associated with a prothrombotic state.

**Materials and methods:**

Patients with moderate (n = 38) and severe periodontitis (n = 30) and controls (n = 24) were recruited. We explored correlations between serum anti-*Aa *and anti-*Pg *IgG and plasma levels of markers of prothrombotic state (von Willebrand Factor [vWF], prothrombin fragment 1+2 [F1+2], plasminogen activator inhibitor-1 [PAI-1] and D-dimer). Multivariate analyses were performed considering several major potential contributing factors.

**Results:**

Periodontitis patients showed higher anti-*Aa *IgG (*p *= 0.015) than controls but not for *Pg *(*p *= 0.320). In periodontitis patients, body mass index and anti-*Aa *IgG showed a positive correlation with vWF (β = 0.297, *p *= 0.010 and β = 0.248, *p *= 0.033 respectively).

**Conclusions:**

In periodontitis, infection with *Aa *together with other well accepted risk factors for CVD, may play a role in increasing the risk for prothrombotic state.

## Introduction

Periodontitis is a chronic infectious disease of the supporting tissues of the teeth and it has been consistently associated with cardiovascular diseases (CVD) [1,2]. One explanation in this association is that periodontitis may also cause a prothrombotic state [3-7]. The prothrombotic state is a propensity of blood to coagulate due to an abnormality in the coagulation and/or fibrinolysis system. In our previous study we measured well established markers of a prothrombotic state which are risk indicators for vascular ischemic events. Prothrombin factor 1+2 (F1+2) is a peptide released during the conversion of prothrombin into thrombin, which is the final step of the coagulation cascade (extrinsic pathway). Von Willebrand Factor (vWF) is expressed by endothelial cells after tissue damage and it triggers aggregation of platelets. Furthermore, vWF is involved in coagulation since it carries the factor VII of the coagulation cascade. Plasminogen activator inhibitor-1 (PAI-1) is an important inhibitor of fibrinolysis and D-dimer is a polymer released during the dissolution of the fibrin clot during fibrinolysis. In our previous study we observed elevated plasma levels of PAI-1 and vWF in periodontitis patients [3].

The systemic dissemination of periodontal pathogens from periodontal lesions seems to be at least one cause for the systemic inflammation in periodontitis and elevation of CVD risk markers. The periodontal pathogens *Aggregatibacter actinomycetemcomitans (Aa) *and *Porphyromonas gingivalis (Pg) *have been shown in blood and biopsies from atherosclerotic plaques [8-10]. IgG and IgA levels against *Aa *and *Pg *have been associated with increased risk of stroke, myocardial infarction and increased carotid artery intima-media thickness as indication for subclinical atherosclerosis [11-14]. Furthermore an *in vitro *study showed that infection with *Pg *can induce a prothrombotic response by increasing the activity of PAI-1. Moreover in a meta-analysis, it was concluded that periodontal disease characterized by elevated markers of bacterial systemic exposure is associated with CVD with a stronger association than clinical parameters of periodontitis [15].

In light of these latter observations, we used in the present pilot study our previous study population and explored whether the association found between periodontitis and a prothrombotic state could be in part explained by the host response to two specific periodontal pathogens. Therefore the aim of this study is to investigate whether in periodontitis serum IgG levels against *Aa *and *Pg *are associated with systemic levels of four markers of a prothrombotic state.

## Materials and methods

### Study population

The study population is retrieved from a previous study [3]. On the basis of an extensive medical history by a written questionnaire and by interview, the following subjects were not included in the study: pregnant women and individuals who suffered from any given disease or chronic medical condition, apart from periodontitis, or had trauma or tooth extractions in the last two weeks, or received antibiotics within the last 3 months. We included all subjects where serum samples were available to determine levels of IgG against *Aa *and *Pg*. Absence of serum sample for several subjects (n = 38) was related to exhaustion of samples in the previous study. All details about recruitment, definition of background variables and approval of Medical Ethical committee are described before. In brief, definition of a periodontal case or a control was based on the 5^th ^workshop guidelines [16], with the modification that for a case > 3 mm proximal bone loss in at least 2 non adjacent teeth needed to be present and for a control subject the distance between the cemento-enamel junction and the alveolar bone crest needed to be < 3 mm on recent bitewing radiographs for all present teeth. We used dental radiographs to estimate the severity of periodontal destruction as described before [17]. Patients with >7 teeth with >50% bone loss were classified as having severe periodontitis. The remainder of the periodontitis patients was classified as having moderate periodontitis.

### Analysis of biochemical background variables and markers of a prothrombotic state

Systemic biochemical factors were retrieved from the data base of our previous study [3]. Background variables included total cholesterol, HDL and LDL cholesterol, triglycerides and high sensitivity CRP (hsCRP). Markers of a prothrombotic state included vWF, prothrombin fragment F1+2, PAI-1 and D-dimer.

### Analyses of serum levels of Immunoglobulin G (IgG) against *Aa *and *Pg*

Serum levels of IgG against two relevant periodontal pathogens, *Aa *and *Pg*, were determined by ELISA as previously described [18], but with a modification [19]. The modification consisted of expanding the antigen mixture, to contain all the known serotypes for *Aa*. For *Aa *strains, ATCC 29523, Y4, NCTC 9710, 3381 and OM2 534 were used, representing respectively serotypes a, b, c, d and e [20]. For *Pg *strains, W83, HG 184, A7A1-28, ATCC 49417, HG 1690, HG 1691 and 34-4 were used, representing respectively the capsule serotypes K1-K7, as well as the uncapsulated strain 381(K^-^) [21].

### Statistical analysis

Statistical analysis of data was performed with the SPSS package version 16.0 (SPSS, Chicago, IL, USA). Means, standard deviations, medians, interquartile ranges and frequency distributions were calculated for background variables, markers of a prothrombotic state and serum IgG's. Normal distribution of data was assessed by Kolmogorov-Smirnov goodness-of-fit test. Whenever non-normal distribution was found (*p *< 0.05), non-parametric tests were employed. Differences for the background variables including markers of prothrombotic state and serum IgG's among the three groups were analyzed by analysis of variance (ANOVA) or Kruskal-Wallis where appropriate. A multivariate analysis (backward stepwise linear regression with *p *= 0.05 to enter and *p *= 0.10 to leave) was performed considering vWF, F1+2, PAI-1 and D-dimer as the outcome variables and using as predictors periodontal status (2 groups [moderate and severe periodontitis]), smoking, education level, age, gender, body mass index (BMI), total cholesterol, triglycerides, ethnicity, anti-*Aa *and anti-*Pg *IgG levels. In the latter explorative parametric tests, for the non-normal distributed variables, the log-transformed values were employed. Similarly, a secondary explorative multivariate analysis was performed, but now including also controls. For all analyses, *p *< 0.05 values were considered statistically significant.

## Results

Table 1 presents the background variables for the current study population derived from Bizzarro et al. (2007). Periodontitis patients were older than controls (45.5 yr for the severe group, 43.8 yr for the moderate group and 38.4 yr for controls, *p *= 0.017) and showed lower educational level (*p *= 0.019). Periodontitis patients had a lower number of teeth (25.7 for the severe group and 26 for the moderate group) in comparison with controls (28.3, *p *= 0.010). Periodontitis patients showed higher levels of PAI-1 in comparison to controls (*p *= 0.009); they also showed higher values for vWF and F1+2 and lower values for D-dimer, but the differences were not statistically significant. IgG levels against *Aa *were significantly higher in patients than controls (*p *= 0.015). Values of IgG against *Pg *show a trend for increased levels in periodontitis (Table 1).

**Table 1:** Characteristics for the 3 study groups (control, moderate and severe periodontitis).

	Control (n = 24)	Moderate Periodontitis (n = 38)	Severe Periodontitis (n = 30)	***p *-value**^**1**^
Age (years)	38.4 ± 11.3	43.8. ± 8.7	45.5 ± 7.7	0.017
				
Gender				NS
Male	7 (29%)	16 (42%)	13 (43%)	
Female	17 (71%)	22 (58%)	17 (57%)	
				
Ethnicity				
Caucasian	21 (88%)	29 (76%)	26 (87%)	NS
Other	3 (12%)	9 (24%)	4 (13%)	
				
Education level				0.019
<High school	6 (25%)	25 (65%)	14 (47%)	
=High school	7 (29%)	2 (5%)	5 (16%)	
>High school	11 (46%)	11 (29%)	11 (37%)	
				
Smoking habits				NS
Smoker	7 (29%)	19 (50%)	14 (47%)	
Non-smoker	17 (71%)	19 (50%)	16 (53%)	
				
BMI (kg/m^2^)	24.6 ± 3.9	26.1 ± 5.5	23.9 ± 2.7	NS
				
Total Cholesterol (mmol/l)	5.2 ± 1.1	5.4 ± 1.2	5.5 ± 1.1	NS
HDL/Chol. (mmol/l)	1.5 ± 0.4	1.3 ± 0.4	1.4 ± 0.4	NS
LDL/Chol. (mmol/l)	3.1 ± 1.0	3.4 ± 1.1	3.6 ± 1.0	NS
				
Triglycerides (mmol/l)	1.2 ± 0.6	1.5 ± 0.8	1.3 ± 0.7	NS
				
Total number of teeth	28.3 ± 2.3	26.0 ± 3.9	25.7 ± 3.2	0.010
Teeth ≥30% bone loss	0.0 ± 0.0	14.2 ± 5.9	21.0 ± 3.5	NT
Teeth ≥50% bone loss	0.0 ± 0.0	2.5 ± 1.6	11.0 ± 4.5	NT
				
hsCRP (mg/l)	1.40 (0.42-4.0)	2.18 (0.92-5.13)	1.43 (0.87-3.13)	NS
				
Prothrombotic state				
vWF (%)	75.0 (63.0-98.5)	92.5 (75.0-111.0)	90.5 (72.7-108.5)	NS
F 1+2 (nmol/l)	0.84 (0.64-1.08)	1.02 (0.82-1.30)	1.01 (0.72-1.36)	NS
PAI-1 (AU/ml)	0.30 (0.20-6.42)	4.32 (0.20-13.60	11.45 (1.47-27.18)	0.009
D-dimer (ng/ml)	31.0 (13.0-68.0)	25.5 (10.5-46.5)	16.0 (1.75-47.2)	NS
				
Serum antibodies				
Anti-*Aa *IgG	0.41 (0.36-0.63)	0.82 (0.45-1.52)	0.85 (0.39-1.58)	0.015
Anti-*Pg *IgG	0.60 (0.49-0.82)	0.66 (0.42-1.07)	0.70 (0.46-1.48)	NS

Values are means ± standard deviations, number of subjects (%) or medians (interquartile ranges).

^1 ^*P*-values obtained by analysis of variance (ANOVA), Kruskal Wallis or Chi squared test, where appropriate.

Assuming that only in periodontitis patients an infection with *Aa *or *Pg *is causing a notable immune response, we explored in periodontitis patients which of the background or systemic variables could be associated with variation in the levels of the markers of a prothrombotic state. Table 2 presents the results per marker the model reached for the periodontitis patients in the multivariate analyses. BMI and IgG levels against *Aa *were significantly associated with levels of vWF (β = 0.297, *p *= 0.010 and β = 0.248, *p *= 0.033 respectively). Female gender and age were significantly associated with F1+2 and a trend for an association between IgG levels against *Pg *and F1+2 (β = 0.227, *p *= 0.058). For PAI-1, severity of periodontitis showed a positive correlation (β = 0.356, *p *= 0.001), but there was no association with IgG against *Aa *and *Pg*. Also for D-dimer both female gender and BMI showed positive correlations, while cholesterol showed a negative correlation; IgG against *Pg *was retained in the final model, with a negative correlation (β = -0.190), but this association did not reach a statistical significance (*p *= 0.085). When the multivariate analyses were repeated including also the control subjects without periodontitis, we observed that the association between IgG against *Aa *and levels of vWF did not reach level of significance (β = 0.181, *p *= 0.072), demonstrating that indeed the exposure to a periodontal pathogen has an association with the prothrombotic marker.

**Table 2:** Multivariate analyses explored for periodontitis patients for markers of a prothrombotic state.

	Subjects N = 68	
	
	**Standardized β**^**1**^	***p-*value**^**1**^
vWF		
BMI	0.297	0.010
Education	- 0.198	0.086
Anti-*Aa *IgG	0.248	0.033
		
F1+2		
Gender	0.258	0.029
Age	0.248	0.039
Anti-*Pg *IgG	0.227	0.058
		
PAI-1		
Severity periodontitis	0.356	0.001
BMI	0.348	0.002
Age	0.283	0.008
Smoking	0.269	0.009
		
D-dimer		
Gender	0.398	0.001
BMI	0.229	0.045
Cholesterol	- 0.267	0.020
Anti-*Pg *IgG	- 0.190	0.085

^1^Standardized correlation coefficient β and the *p*-values obtained from a multivariate analysis (backward step-wise linear regression with *p *= 0.10 to enter and *p *= 0.05 to leave) using vWF, F1+2, PAI-1, D-dimer as dependent variables and age, gender (male = 0 and female = 1), ethnicity (not Caucasian = 0 and Caucasian = 1), smoking status (non-smoker = 0 and smoker = 1), educational level (3 groups), BMI, total cholesterol, triglycerides, anti-*Aa *IgG levels, anti-*Pg *IgG levels and severity of periodontitis (2 groups, moderate and severe periodontitis) as predictors. The retained predictors in the final models are presented.

## Discussion

In a previous study, our group showed that systemically healthy periodontitis patients, in comparison to subjects without periodontitis, displayed higher levels of 2 markers of prothrombotic state, PAI-1 and vWF [3]. Based on these results, our aim was to investigate whether, in the same population of periodontitis patients, IgG levels against *Aa *and *Pg *were explanatory for these findings.

This question was raised by the evidence in the literature, which shows that levels of IgG against *Aa *and *Pg *have been associated with CVD. In particular, a 13-year prospective study from Pussinen et al. (2004) showed a strong association between anti-*Aa *and anti-*Pg *IgG's at baseline and the development of stroke later on. Furthermore *Aa *and *Pg *have been found in human specimens of atherosclerotic plaques [8-10]. However, we have no clinical evidence about the possible association between *Aa *and *Pg *with a prothrombotic state. So far, only *in vitro *or *ex vivo *studies are available. More specifically, *Aa *showed to have capability to induce *ex vivo *platelet aggregation in humans [5], while *Pg *showed capability to induce *in vitro *coagulation [22] and to enhance PAI-1 activity after *in vitro *infection of aortic endothelial cells with the *Pg *strain 381 [23].

The main finding of the current study was that in patients affected by periodontitis, in the multivariate analysis levels of IgG against *Aa *correlated with levels of vWF after correction and including several other potential confounding factors. Notably, this association was not longer significant when we included in the study population also the controls. This strengthens the assumption that in periodontitis patients, where the exposure to the periodontal pathogens is stronger, infection with *Aa *may trigger a prothrombotic effect. In fact, this effect seems to be diluted by adding in our multivariate model subjects without periodontitis and with a relatively low response to *Aa*. We confirm that also BMI, age, female gender, smoking and cholesterol play an important role [24]. Nevertheless, these results underline a possible role of infection with periodontal pathogens in periodontitis patients, where the immune response to these periodontal bacteria is enhanced. vWF plays an important role in inducing coagulation since it is a carrier of coagulation factor VII and, when expressed by endothelial cells, it induces platelet adhesion to the endothelium. Although we cannot give a biological explanation of the possible role of *Aa *in the mechanism that may provoke a prothrombotic state, we may speculate that the lipopolysaccharide (LPS) produced by *Aa *may trigger the endothelial cells to produce more vWF [25] or, alternatively, *Aa *itself may invade endothelial cells and then stimulate the release of vWF [26].

In contrast with the findings of Roth et al. (2006), we did not find a correlation between PAI-1 activity and serum IgG against *Pg*. An explanation could be the fact the Roth et al. studied selectively the production of PAI-1 by endothelial cells *in vitro*; however, *in vivo*, PAI-1 is produced not only by endothelial cells but also in the liver and by adipocytes. Yet, in our multivariate analysis we found in periodontitis patients a trend for a positive correlation between F1+2 and IgG levels against *Pg *and an inverse correlation between levels of IgG against *Pg *and levels of D-dimer. F1+2 is a peptide formed during the conversion of prothrombin into thrombin in the coagulation cascade and D-dimer is a marker of the turnover of fibrin dissolution in fibrinolysis, the process of breakdown of the blood clot. This might suggest that *Pg *may play some role in enhancing coagulation and suppressing fibrinolysis. These suggestions need to be confirmed in further research with larger populations.

There are some limitations in this study that need to be discussed. As already mentioned, this study population was retrieved from the population of our previous investigation. Now we determined the IgG levels against *Aa *and *Pg*. Unfortunately, for a number of the original study subjects these measurements could not be performed because of exhaustion of available serum. Lack of serum for some individuals (both patients and controls) led to the exclusion of these subjects and as a consequence a reduction in the population size, in comparison to the original population. Another point of discussion is the lack of information about subgingival presence of *Aa *and *Pg *in the study population. However we consider the levels of IgG against *Aa *and *Pg *as a reliable indicator of a possible infection with these periodontal pathogens. This is currently a widely used method in the literature [11,12,14]. Nevertheless, preliminary experiments on a group of 19 untreated periodontitis patients showed a very good correlation between the measured levels of anti-*Aa *IgG and the number of colony-forming units of *Aa *cultured from subgingival flora (r = 0.800, p < 0.001). Similar results were obtained for *Pg *(r = 0.650, p = 0.003).

## Conclusion

In conclusion, this pilot study confirms that well accepted risk factors for CVD correlate with markers of prothrombotic state; these include BMI, gender, age, smoking and cholesterol. However we also propose that at least periodontal infection with *Aa *may also play an important role, while infection with *Pg *is suggestive. Further research will be necessary to confirm the findings of the current study. In general it needs to be considered that bacterial infection in addition to traditional parameters may also contribute to a prothrombotic state.

## List of abbreviations

BMI: Body Mass Index; hsCRP: high sensitive C-reactive protein; NS: not significant; NT: not tested; vWF: von Willebrand factor; PAI-1: plasminogen activator inhibitor-1; F1+2: prothrombin fragment 1+2; IgG: Immunoglobulins G; AU: Arbitrary Units.

## Conflict of Interest and source of funding statement

The authors declare that there are no conflicts of interest in this study. This study was supported in part by the author's institution and in part by Philips Oral Healthcare EMEA.

## Authors' contributions

SB participated in the design, the conduction of the study and data collection, performed the statistical analysis and he is the main author of the manuscript. EAN researched, designed and carried out the ELISA's on IgG's against *Aa *and *Pg*. UvdV provided intellectual contribution in the interpretation of the data and participated in drafting the manuscript. MLL assisted in ELISA's and the statistical analyses and helped to draft the manuscript. BGL participated substantially in the design, analyses and interpretation of data and participated in drafting the manuscript.

All authors read and approved the final manuscript.
